# Editorial: Aphids as plant pests: from biology to green control technology

**DOI:** 10.3389/fpls.2023.1337558

**Published:** 2024-01-08

**Authors:** Julian Chen

**Affiliations:** State Key Laboratory for Biology of Plant Diseases and Insect Pests, Institute of Plant Protection, Chinese Academy of Agricultural Sciences, Beijing, China

**Keywords:** aphid, endosymbiont, natural enemies, plant resistance to aphid, chemical ecology, green control method, IPM (integrated pest management)

The Research Topic “*Aphids as Plant Pests: From Biology to Green Control Technology*” was edited by Professor Julian Chen from the Institute of Plant Protection, Chinese Academy of Agricultural Sciences; Professor Leonardo A. Crespo-Herrera from International Maize and Wheat Improvement Center (CIMMYT); and Professor Frederic Francis from the University of Liege, as guest editors during 2022 and 2023.

The aim and objective of the Research Topic was to bring together high-quality articles from researchers working on the area of aphids (Hemiptera: Aphidoidea), which are the most important and destructive plant pests. The Research Topic includes material on interactions between aphids and plants, the natural enemies and endosymbionts of aphids. The focus of each of the articles and reviews targets different aspects of the relevant biology and ecology, molecular-level issues, and interactions with agricultural factors, including novel strategies for green control of these insect pests and alternatives to chemical control.

This Research Topic has been produced in collaboration with *Frontiers in Plant Science*. We announced and published the Research Topic on July 14, 2022, and manuscript submissions ended on January 14, 2023. A total of 17 manuscripts were submitted, of which 10 articles were accepted, consisting of one review and nine original research articles. All accepted articles have been published in Open Access form. Additionally, we aim to put together a free Research Topic of all published manuscripts to provide an up-to-date and comprehensive overview of the latest research progress in the field. The main findings on aphids can be summarized under the following categories: from identification to forecasting, and potential global distribution in the context of climate change; the population dynamics of aphid and their symbionts; molecular biology for development, metabolism, and host adaptation; and management techniques, e.g., RNAi, ecological and biological control, and integrated pest management (IPM).

## 1 From identification to forecasting, and projections of potential distribution

Manual identification and quantitative analysis of trap catches are necessary in classical collection methods. Machine learning, image recognition, and artificial intelligence are useful methods for the identification of food-contaminating beetle species (Wu et al., 2019). Batz et al. proved that such methods could be used for small insect pests: specifically, for systematic monitoring of aphids, automated identification, and intelligent forecasting.

Climate change impacts crop production and the interaction of biotic and abiotic stresses, posing considerable threats to sustainable food security (Wang et al., 2022; Robles-Zazueta et al., 2023). Warming of the climate affects biological invasions by shifting interactions between plants and herbivores (Lu et al., 2013); this highlights the importance of assessments of the effects of climate warming on the population distributions of pests and their natural enemies. Lian et al. analyzed key environmental factors affecting both the survival and the potential distribution of *Lipaphis erysimi*, an important vegetable aphid, and its dominant predatory natural enemy, the hoverfly *Eupeodes corollae*, based on the MaxEnt model and using data on the geographical distribution of historical occurrences. Their data will be beneficial for pest-monitoring and for early warning and biological control systems.

## 2 Population dynamics of aphids and symbionts

A suction trap is useful method of surveying migration in small insect pests, such as aphids. Wheat aphids, in particular, are migrant pests. Using data from suction traps, Li et al. analyzed the migration patterns of the wheat aphid species *Sitobion miscanthi*, *Rhopalosiphum padi*, and *Schizaphis graminum* in the northern plains of the wheat-growing region of China during the period 2018 to 2020. Aphid migration trajectories changed over the years, as simulated by the NOAA HYSPLIT model. The facultative bacterial symbionts of migrant wheat aphids were investigated with specific PCR and amplicon sequencing. The dominant infection strains (*Serratia symbiotica*, *Hamiltonella defensa*, and *Regiella insercticola*) were identified from *S.miscanthi*, *R. padi*, and the diversity of the bacterial community varied across wheat aphid species and migrant populations.

## 3 Molecular biology for development, metabolism, and host adaptation

Omics approaches, i.e., transcriptomes, proteomes, secretomes, and so on, are useful methods of understanding insect development, metabolism, and host adaptation.

Taking the horned gall aphid *Schlechtendalia chinensis* as a test target, Wei et al. combined data from morphological observations of male aphids using SEM, high-throughput transcriptome sequencing, and weighted gene co-expression network analysis; they identified gene co-expression modules and hub genes correlated with body size traits and speculated that the male aphid degrades its own substances via autophagy and apoptosis in order to sustain life activities, resulting in body size reduction after molting.

In aphids with piercing–sucking mouth parts, salivary glands and their secretions play an important role in the feeding process. The identification of saliva proteins is an important step in understanding the adaptation of aphids to host plants (Zhang et al., 2017; Zhang et al., 2022; Zhang et al., 2023). Zhang et al. selected *Pseudoregma bambucicola*, a severe bamboo pest, as the target of their analysis; this aphid species feeds on stalks of bamboos by degrading hard bamboo cell walls. Using salivary transcriptome, secretome, and proteome analyses, they detected some secretory proteins homologous to known aphid effectors in *P. bambucicola*. Although plant cell wall-degrading enzymes were identified, most of them were expressed at low levels in the aphid salivary glands. These results imply that symbiotic bacteria of *P. bambucicola* may help the aphid adapt to a specific habitat.

Aphids and their primary symbiont, *Buchnera aphidicola*, form a stable and mutually beneficial relationship that plays an important role in providing the necessary nutrients to the host aphid (Li et al., 2023). Based on the genome sequence of the wheat aphid *Siotobion miscanthi* and its primary symbiont *Buchnera*, which the authors had previously studied, Li et al. identified a metabolic relay gene, ilvA, linking the aphid and *Buchnera* in the isoleucine synthesis pathway, with higher levels of expression observed in the aphid bacteriocytes. This gene is also conserved in different aphid genomes. The function of ilvA is important in aphid development and reproduction, as evaluated through RNAi.

## 4 Management techniques

### 4.1 RNAi

RNA interference (RNAi) targeting an insect-specific gene is an important method of evaluating gene function and plays a role in pest management. For aphid gene knockdown, RNAi is usually conducted using molecules of double-stranded RNA (dsRNA) via an artificial diet or plant-mediated expression. Zhang et al. identified and characterized a novel potential RNAi target gene (*SmDSR33*) encoding a salivary protein in grain aphids (*Sitobion miscanthi*). Through plant-mediated RNAi, transgenic wheat lines expressing dsRNA for targeted silencing of *SmDSR33* were developed, and were found to significantly reduce aphids’ fecundity, survival, and feeding behavior. *SmDSR33*, an essential salivary protein gene, could be exploited as an effective RNAi target through plant-mediated RNAi for aphid control in wheat.

### 4.2 Ecological and biological control

Infochemicals are important cues in the interactions of plants, herbivores and their natural enemies. Some herbivore-induced plant volatiles (HIPVs) are key components in attracting natural enemies, which is also beneficial for the development of techniques for ecological and biological control. Xu et al. examined an orchard ecosystem, including *Vitex negundo* (Lamiaceae), an indigenous plant species in northern China; the cotton aphid *Aphis gossypii*; and the predatory ladybug *Harmonia axyridis.* The candidate active HIPVs mediated by the aphid, including sclareol, eucalyptol, nonanal, and a-terpineol, may attract *H. axyridis*, indicating a potential avenue for improving biological control of aphids in orchards.

Agrosystem biodiversity benefit pest management: based on the natural enemy hypothesis, a complex environment favors natural enemies, e.g., banker plant systems using non-pest arthropod species as alternative prey. In order to select the right banker plants in IPM programs, it is necessary to acquire a deep understanding of the indirect interactions between the target pest and alternative prey, as mediated by biocontrol agents. Wang et al. established and studied a banker plant system, including a banker plant (*Vicia faba*), an alternative prey (*Megoura japonica*), and a predator (*Harmonia axyridis*), to control the target pest (*Myzus persicae*) on pepper. The presence of the alternative prey was able to increase *H. axyridis* population and provide benefits for the targeted aphid control.

### 4.3 IPM

Integrated pest management (IPM) is an environmentally sustainable approach that involves using a combination of practices and control methods to manage a pest at low cost with minimal use of chemical pesticides. The demonstration of technologies providing effective control is important in the evaluation of pest management systems. Uyi et al. targeted *Melanaphis sorghi*, a serious pest of the sorghum plant in the southern USA, and investigated the effects of host plant resistance (DKS37-07) deploy pesticide application, and biological control of natural enemies on *M. sorghi* in five sorghum production locations in four states in the southeastern USA. The integration of these three components is central to the development of *M. sorghi* management.

## Author contributions

JC: Writing – original draft, Writing – review & editing.
